# Supplementary material for: Kin Selection in the RNA World
Source: Life (Basel). 2017 Dec 5;7(4):53. doi: 10.3390/life7040053 (PMC5745566; doi:10.3390/life7040053)
Supplement: Supplementary file 1 [file life-07-00053-s001.pdf]

In this supplementary information, we do three things: derive the fitness function Equation (10) used in the main paper; calculate the ESS for the fitness function in Equation (10); more generally, demonstrate the method in solving for an ESS and 'closing a model' (writing relatedness in terms of model parameters).

0.1 Deriving the Fitness Function in the Text

Here, we derive the fitness function in Equation (10). If an individual survives (with probability k), its fitness is 1; otherwise, with probability $(1-k)$, its fitness is equal to its offsprings' fitnesses. A proportion d compete globally, and therefore have fitness relative to the global average, which we assume to be 1 (the population is neither growing nor shrinking). A proportion $1-d$ remain locally, and their fitness is relative to the average fitness in the social group. After diffusion, the number of individuals on a patch is equal to the number of individuals produced on a patch that stay (with probability $1-d$) plus the number of individuals arriving from elsewhere (dN) ([?]). So, the total number of offspring on a patch after diffusion is:

$$(1-d)\left[N+N\left(bZ\left(1-Z\right)-cZ\right)\right]+dN = N[1+(1-d)\left(bZ\left(1-Z\right)-cZ\right)]. \quad (1)$$

From this, we can write the fitness function in the text, Equation (10):

$$\begin{aligned} w\left(x,y,Z\right) = (1-k)\left(d\right)\left(x\left(1-c+by\right)+\left(1-x\right)\left(1+by\right)\right)+ \\ (1-d)\frac{x\left(1-c+by\right)+\left(1-x\right)\left(1+by\right)}{1+\left(1-d\right)\left(bZ\left(1-Z\right)-cZ\right)}+k. \end{aligned} \quad (2)$$

0.2 Calculating the ESS

To calculate the ESS for Equation (10), we use the Taylor–Frank approach. However, the solution is not analytically tractable. Instead, if we assume b and c to be small, we can write a first-order approximation of Equation (10) as

$$w\left(x,y\right)=\left(1-k\right)\left[1-cx+\left(1-x\right)by-\left(bZ\left(1-Z\right)-cZ\right)\left(1-d\right)^{2}\right]+k. \quad (3)$$

We previously showed that relaxing the assumption of small b and c does not qualitatively alter the results ([**?**]). From this, we can use the Taylor–Frank (1996) approach to solve for the ESS ([**?**]. We take the derivative of fitness with respect to phenotype, solving for $x = y = x^*$, and candidate ESSs occur where:

$$\begin{aligned}\frac{dw}{dg}&=\frac{\partial w}{\partial x}+R\frac{\partial w}{\partial y}\\&=-c-bx-(1-d)^{2}\left(-\left(\frac{c}{n}\right)-\frac{\left(b\left(x+\left(-1+n\right)x\right)\right)}{n^{2}}+\frac{b\left(1-\frac{x+\left(-1+n\right)x}{n}\right)}{n}\right)+\\&r*\left(b\left(1-x\right)-\left(1-d\right)^{2}\left(-\frac{c\left(-1+n\right)}{n}-\frac{b\left(-1+n\right)\left(x+\left(-1+n\right)x\right)}{n^{2}}+\right.\right.\\&\left.\left.\frac{b\left(-1+n\right)\left(1-\frac{x+\left(-1+n\right)x}{n}\right)}{n}\right)\right)\\&=0.\end{aligned} \quad (4)$$

Solving for x^*, we get:

$$x^* = \frac{cN - bNR + b(1-d)^2 - c(1-d)^2 - bR(1-d)^2 + cR(1-d)^2 + bNR(1-d)^2 - cNR(1-d)^2}{b\left(-N - NR + 2(1-d)^2 - 2R(1-d)^2 + 2NR(1-d)^2\right)}, \tag{5}$$

which is the ESS value of cooperation.

0.3 Writing Relatedness in Terms of Model Parameters

Equation (S5) gives the ESS in terms of R and other model parameters, but we expect R to depend on d, k, and N. Here, we calculate R in terms of those parameters, though more generally any parameters may impact R, and the following approach readily extends to such cases. We start by determining the relatedness, at equilibrium, of a focal RNA molecule to a random molecule in its social group, including itself. This is known as whole-group relatedness (denoted by $\mathbb{R}$), because it includes the focal individual, in contrast to others-only relatedness (R), which does not include the focal individual ([**?**]). Our model requires others-only relatedness, because y is the average of the individuals on the patch, excluding the focal individual. $\mathbb{R}$ is the relatedness between two individuals drawn randomly from a local group with replacement. We can write this as the probability that those two individuals are the same individual ($1/N$), and thus have relatedness 1, plus the probability that those two individuals are not the same ($(N-1)/N$), and thus have the relatedness of two random individuals drawn without replacement, or others-only relatedness, R:

$$\mathbb{R} = \frac{1}{N} + \frac{N-1}{N}R \tag{6}$$

Equation (S6) is a general equation for relatedness, for any infinite population of individuals subdivided into N social groups. Now we take two individuals (without replacement) on the same patch with relatedness R, and determine the

relatedness of their representatives in the previous generation. With chance k^2, they are both survivors from the previous generation, in which case their relatedness is the same (R). With chance $2k(1-k)$, one is a survivor and the other is a new offspring, which is native with probability $(1-d)$, in which case their relatedness is $\mathbb{R}$. Otherwise, with chance $(1-k)^2$, they are both new offspring, are both native with probability $(1-d)^2$, and thus have relatedness $\mathbb{R}$. Others-only relatedness between two individuals in the current generation is equal to

$$R_t = k^2 R_{t-1} + 2k\,(1-k)\,(1-d)\,\mathbb{R}_{t-1} + (1-k)^2\,(1-d)^2\,\mathbb{R}_{t-1} \tag{7}$$

Here, R_t is relatedness in the current generation, or time step, and R_{t-1} and $\mathbb{R}_{t-1}$ are others-only and whole-group relatednesses, respectively, in the previous one. Note that Equation (S7) was derived assuming that the only population processes affecting relatedness were survival and diffusion, but a similar recursion could be written taking into account any parameters that affect relatedness. Setting $R_t = R_{t-1}$, we find the equilibrium others-only relatedness. Plugging into equation (A5), we find the equilibrium value of whole-group relatedness, $\mathbb{R}^*$, to be:

$$\mathbb{R}^* = \frac{1+k}{N + kN + 2k\,(1-d) - 2kN\,(1-d) + (1-d)^2 - k\,(1-d)^2 - N\,(1-d)^2 + kN\,(1-d)^2} \tag{8}$$

This equation for relatedness was identified by Taylor and Irwin 2000 ([**?**]). However, our model in Equation (10) in the text is modelling an others only-trait, and thus requires others-only relatedness, R. $\mathbb{R}N$ gives us the number of relatives on our patch. Subtracting the focal individual, and dividing by the total number of remaining individuals $(N-1)$, gives us R^*:

$$R^* = \frac{\frac{N(1+k)}{(1+k)N-(N-1)(2k+(1-d)-k(1-d))(1-d)} - 1}{N-1} \tag{9}$$

Plugging into Equation (S5) gives us Equation (11) in the text.
